# Supplementary material for: Perinatal factors affect the gut microbiota up to four years after birth
Source: Nat Commun. 2019 Apr 3;10:1517. doi: 10.1038/s41467-019-09252-4 (PMC6447568; doi:10.1038/s41467-019-09252-4)
Supplement: Supplementary file 1 — Supplementary Information [file 41467_2019_9252_MOESM1_ESM.pdf]

# Microbiome Memory of perinatal factors that affect the gut microbiota four years after birth

Fouhy F & Watkins C *et al.*

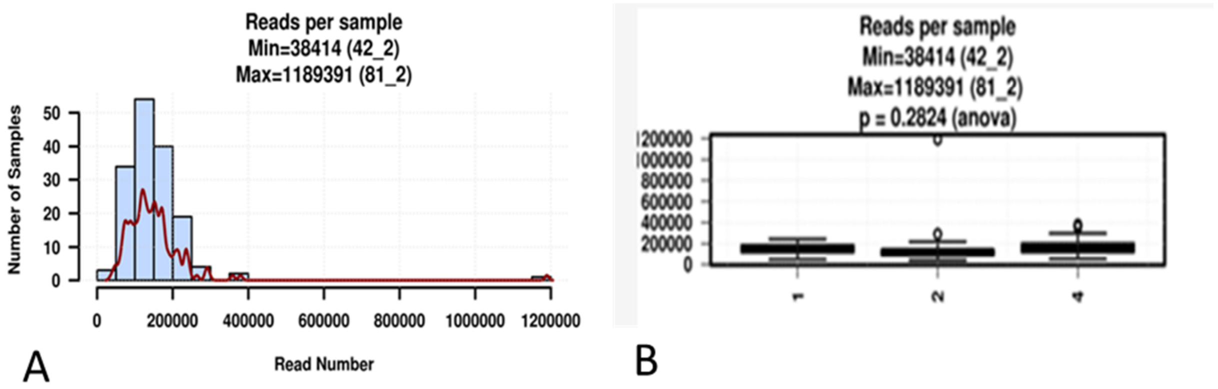


**Supplementary Fig.1: A** Numbers of reads per sample generated using 2x250bp Illumina MiSeq sequencing; **B** number of sequencing reads per sample by year of infant (1, 2 and 4)

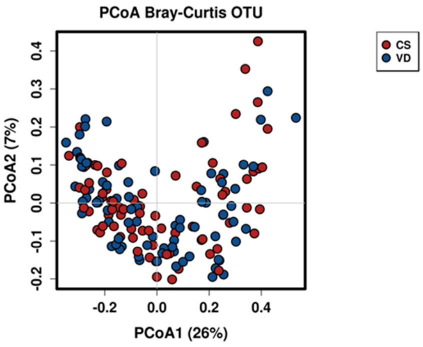


**A) Mode of Delivery**

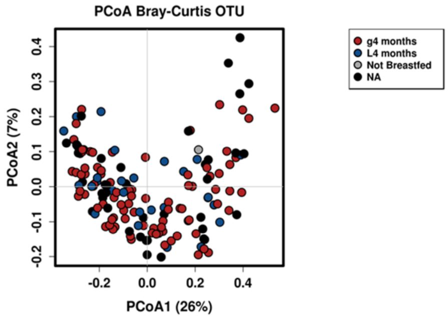


**B) Breastfeeding duration**

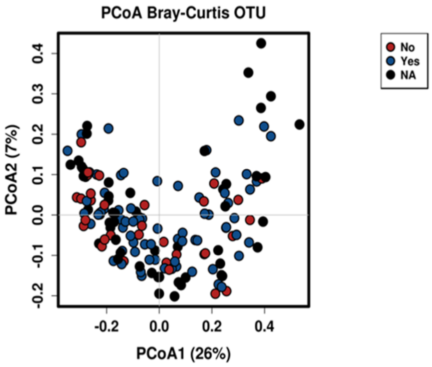


**C) Antibiotics taken during first year of life**

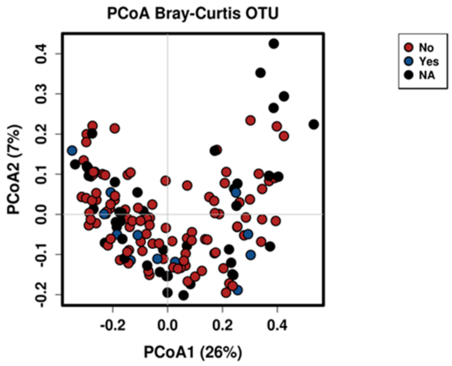


**D) Probiotic exposure in first year of life**

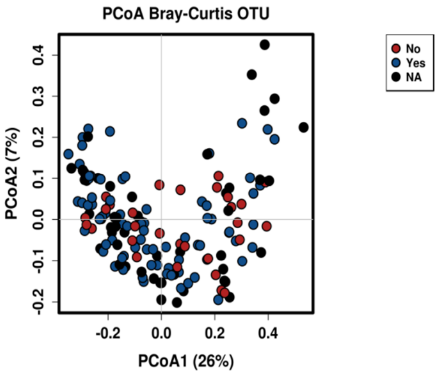


**E) Other Siblings**

**Supplementary Fig.2**: PCoA analysis using Bray-Curtis distance matrices based on; **A** Mode of delivery, **B** breastfeeding duration (g > 4, L < 4 months); **C** antibiotics taken during first year of life; **D** probiotic exposure in first year of life; **E** other siblings

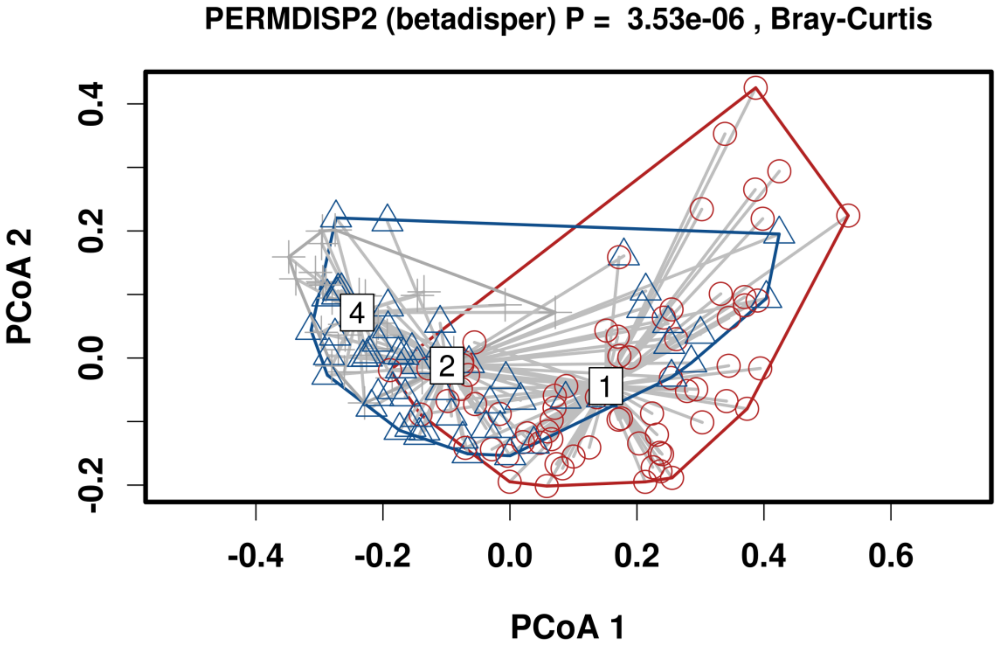


**Supplementary Fig. 3:** Microbiota composition was significantly different between groups based on year using PERMDISP2 permutational analysis. P-value is calculated based on distances of each sample to the group centroid in the PCoA and describes significance of the grouping

**A) Year one – Gestational age at birth.**

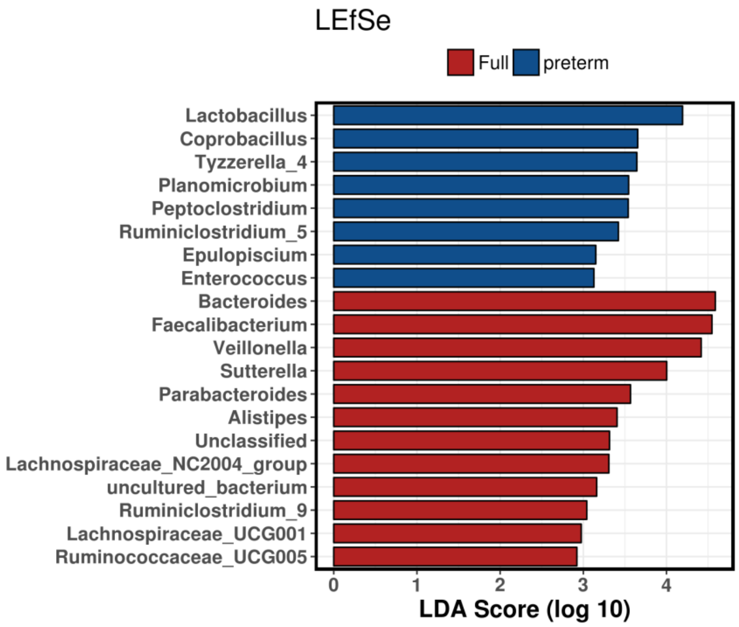


**B) Year two – Gestational age at birth.**

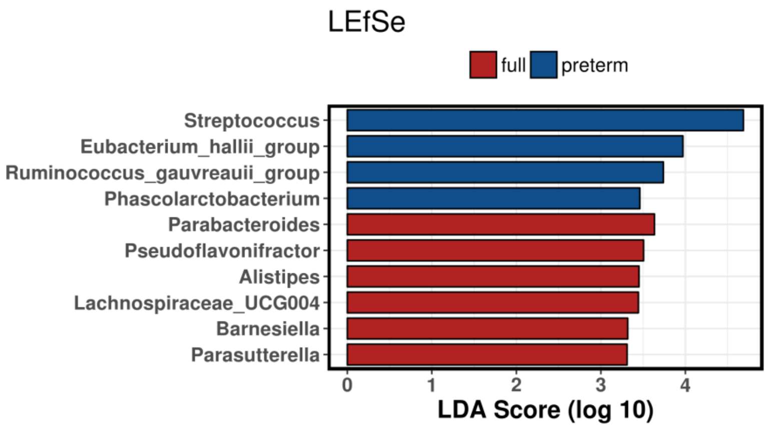


**C) Year four – Gestational age at birth.**

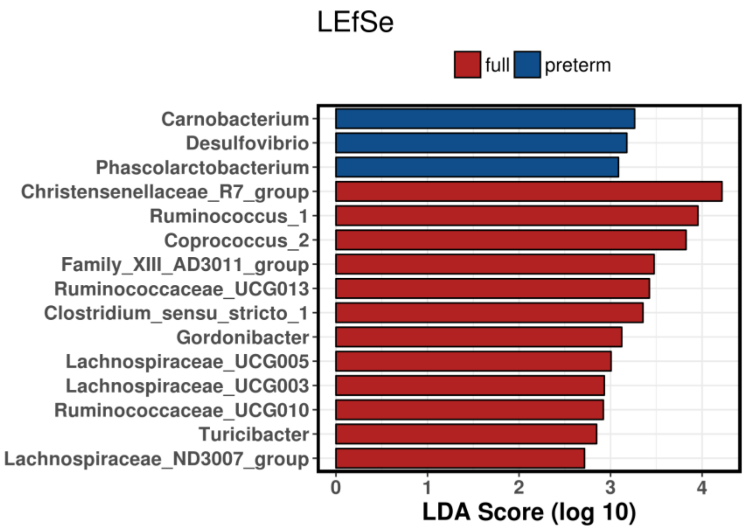


**Supplementary Fig. 4:** LDA Effect Size (LEfSe) analysis on gut microbiota composition based on gestational age at birth at A) year one, B) year two and C) year four

**A) Year one – Delivery mode.**

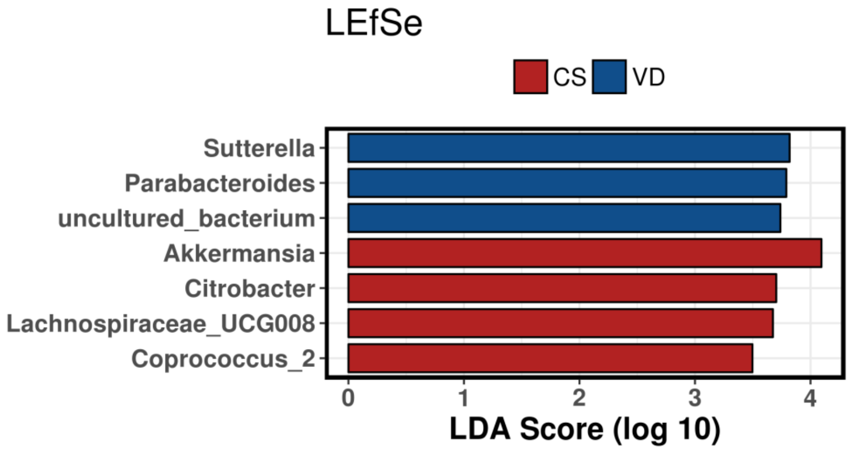


**B) Year two – Delivery mode.**

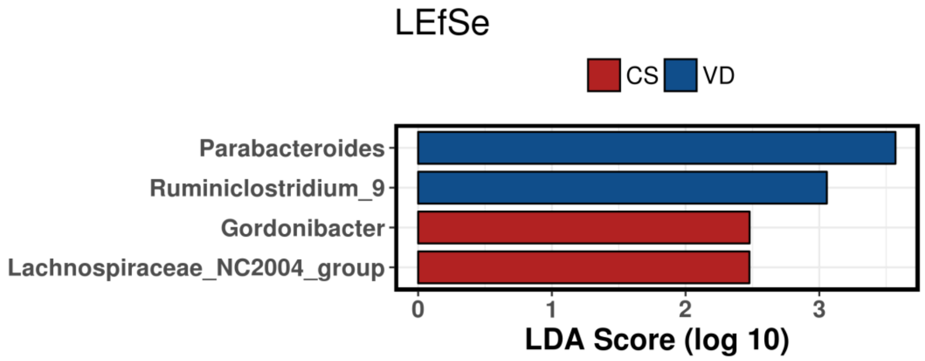


**C) Year four – Delivery mode.**

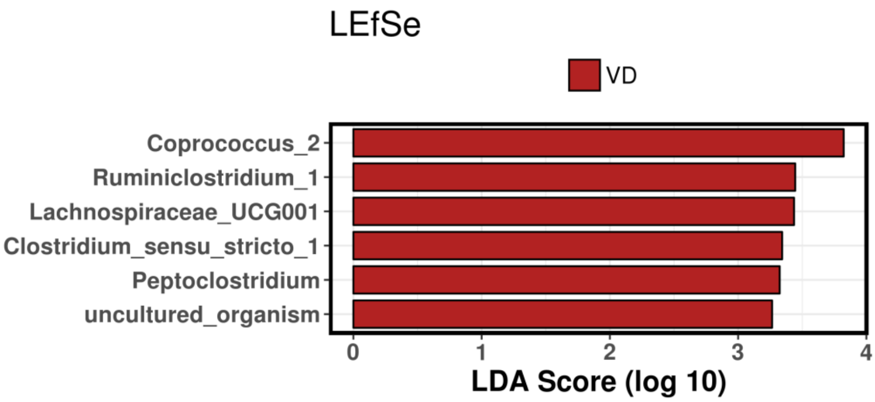


**Supplementary Fig. 5:** LDA Effect Size (LEfSe) analysis on gut microbiota composition based on mode of delivery at A) year one, B) year two and C) year four
