# Supplementary material for: Perinatal factors affect the gut microbiota up to four years after birth
Source: Nat Commun. 2019 Apr 3;10:1517. doi: 10.1038/s41467-019-09252-4 (PMC6447568; doi:10.1038/s41467-019-09252-4)
Supplement: Supplementary file 3 — Description of Additional Supplementary Files [file 41467_2019_9252_MOESM3_ESM.docx]

**Description of Supplementary Files**

**File Name:** Supplementary Data 1.

**Description:** Total number of infant faecal samples collected at years 1, 2 and 4. Children had been born by caesarean section (CS) or vaginal delivery (VD).

**File Name:** Supplementary Data 2.

**Description:** Metadata infant participants.

**File Name:** Supplementary Data 3.

**Description:** Repeated measures statistical analysis based on year adjusting for gestational age.
