# Supplementary material for: Perinatal factors affect the gut microbiota up to four years after birth
Source: Nat Commun. 2019 Apr 3;10:1517. doi: 10.1038/s41467-019-09252-4 (PMC6447568; doi:10.1038/s41467-019-09252-4)
Supplement: Supplementary file 7 — Reporting Summary [file 41467_2019_9252_MOESM7_ESM.pdf]

nature research

Corresponding author(s): Prof. Catherine Stanton

Last updated by author(s): 20/02/2019

Reporting Summary

Nature Research wishes to improve the reproducibility of the work that we publish. This form provides structure for consistency and transparency in reporting. For further information on Nature Research policies, see Authors & Referees and the Editorial Policy Checklist.

Statistics

For all statistical analyses, confirm that the following items are present in the figure legend, table legend, main text, or Methods section.

n/a	Confirmed	
☐	☒	The exact sample size (*n*) for each experimental group/condition, given as a discrete number and unit of measurement
☐	☐	A statement on whether measurements were taken from distinct samples or whether the same sample was measured repeatedly
☐	☒	The statistical test(s) used AND whether they are one- or two-sided<br>*Only common tests should be described solely by name; describe more complex techniques in the Methods section.*
☐	☒	A description of all covariates tested
☐	☒	A description of any assumptions or corrections, such as tests of normality and adjustment for multiple comparisons
☐	☒	A full description of the statistical parameters including central tendency (e.g. means) or other basic estimates (e.g. regression coefficient) AND variation (e.g. standard deviation) or associated estimates of uncertainty (e.g. confidence intervals)
☐	☒	For null hypothesis testing, the test statistic (e.g. *F*, *t*, *r*) with confidence intervals, effect sizes, degrees of freedom and *P* value noted<br>*Give P values as exact values whenever suitable.*
☒	☐	For Bayesian analysis, information on the choice of priors and Markov chain Monte Carlo settings
☒	☐	For hierarchical and complex designs, identification of the appropriate level for tests and full reporting of outcomes
☒	☐	Estimates of effect sizes (e.g. Cohen's *d*, Pearson's *r*), indicating how they were calculated

Our web collection on statistics for biologists contains articles on many of the points above.

Software and code

Policy information about availability of computer code

Data collection	No software was used for collection of clinical data. This was provided by the designated research nurse on the project.
Data analysis	FLASH (FLASH: fast length adjustment of short reads to improve genome assemblies), removal of mismatched barcodes and sequences was completed using QIIME(1), OTU grouping were performed using USEARCH v7, OTUs were aligned using PyNAST and taxonomy was assigned using BLAST against the SILVA SSURef database release 123. Statistical analysis was performed using the Calypso online software (version 8.68).

For manuscripts utilizing custom algorithms or software that are central to the research but not yet described in published literature, software must be made available to editors/reviewers. We strongly encourage code deposition in a community repository (e.g. GitHub). See the Nature Research guidelines for submitting code & software for further information.

Data

Policy information about availability of data

All manuscripts must include a data availability statement. This statement should provide the following information, where applicable:

- Accession codes, unique identifiers, or web links for publicly available datasets
- A list of figures that have associated raw data
- A description of any restrictions on data availability

The data that supports the findings of this study are available upon reasonable request from the corresponding author.

Field-specific reporting

Please select the one below that is the best fit for your research. If you are not sure, read the appropriate sections before making your selection.

☒ Life sciences ☐ Behavioural & social sciences ☐ Ecological, evolutionary & environmental sciences

For a reference copy of the document with all sections, see nature.com/documents/nr-reporting-summary-flat.pdf

Life sciences study design

All studies must disclose on these points even when the disclosure is negative.

Sample size	Fresh faecal samples were collected from 70 infants at year one, 57 at year two and 32 at year four, based on previously published infant gut microbiota studies of similar size.
Data exclusions	No data was excluded from this study.
Replication	Three sequencing runs were carried out. A number of samples were repeated on each sequencing run to ensure the reproducibility of the sequencing data.
Randomization	No randomisation occurred as the the groups were based on the age of the individual, i.e samples at year one, two and four.
Blinding	The investigators were aware of the age of the individuals being tested.

Reporting for specific materials, systems and methods

We require information from authors about some types of materials, experimental systems and methods used in many studies. Here, indicate whether each material, system or method listed is relevant to your study. If you are not sure if a list item applies to your research, read the appropriate section before selecting a response.

Materials & experimental systems

n/a	Involved in the study	
☒	☐	Antibodies
☒	☐	Eukaryotic cell lines
☒	☐	Palaeontology
☒	☐	Animals and other organisms
☐	☒	Human research participants
☒	☐	Clinical data

Methods

n/a	Involved in the study	
☒	☐	ChIP-seq
☒	☐	Flow cytometry
☒	☐	MRI-based neuroimaging

Human research participants

Policy information about studies involving human research participants

Population characteristics	Fresh faecal samples were collected from 70 infants at year one (43% female), 57 at year two and 32 at year four from the Cork University Maternity Hospital, Cork, Ireland, as part of the INFANTMET study.
Recruitment	Briefly, mothers were approached between February 2012 and May 2014 and asked for consent at Cork University Maternity Hospital.
Ethics oversight	Ethical approval was provided by the Cork University Hospital Research Ethics Committee (reference ECM (w) 07/02/2012).

Note that full information on the approval of the study protocol must also be provided in the manuscript.
